# The Prevalence and Factors Associated With Antipsychotic Polypharmacy in a Forensic Psychiatric Sample

**DOI:** 10.3389/fpsyt.2020.00263

**Published:** 2020-04-17

**Authors:** Christian Farrell, Johann Brink

**Affiliations:** Faculty of Medicine, University of British Columbia, Vancouver, BC, Canada

**Keywords:** antipsychotics, polypharmacy, forensic, prescribing practices, psychiatry, inpatients

## Abstract

Despite clinical guidelines limiting the use of multiple concomitant antipsychotics to the most exceptional and treatment resistant cases, the prevalence of antipsychotic polypharmacy has been increasing worldwide. There has been minimal research investigating the prevalence of antipsychotic polypharmacy in forensic psychiatric samples and the correlates associated with antipsychotic polypharmacy. This cross-sectional study aimed to establish the prevalence of antipsychotic polypharmacy in a forensic psychiatric inpatient sample and to investigate the demographical, clinical, and forensic factors associated with polypharmacy. All patients (*N =* 142) were prescribed at least one antipsychotic at the time of the study. Antipsychotic polypharmacy was prescribed to 54.93% of patients. Logistic regression results indicated increased length of hospitalization, high/medium security level, treatment with clozapine, and depot antipsychotic prescription were predictive of being placed on an antipsychotic polypharmacy regimen. The results suggest that those who are prescribed multiple antipsychotics are long stay patients who present with higher clinical complexity. The results from this study can be used to inform clinical practice leaders about the prevalence of antipsychotic polypharmacy in a forensic psychiatric institution. More research is needed to understand the clinical justifications for prescribing multiple antipsychotics in a forensic psychiatric sample and ways to safely reduce the prevalence of antipsychotic polypharmacy.

## Introduction

Current Canadian guidelines in the treatment of individuals with schizophrenia advise limiting the use of multiple concurrent antipsychotics to the most exceptional and treatment resistant cases (1). Despite minimal evidence supporting the use of antipsychotic polypharmacy, it remains a common practice worldwide (1–4). For example, Yang and colleagues (5) investigated the prevalence of antipsychotic polypharmacy in 15 countries in Asia and found the average rate of antipsychotic polypharmacy was 42.2%. A recent study in the United States found a prevalence of 27.2% (6). In fact, Gallego and colleagues identified a 34% increase in the prevalence of antipsychotic polypharmacy in North America from the 1980s to 2000s (3). Additionally, antipsychotic polypharmacy is prevalent in a variety of psychiatric settings, including outpatient, inpatient, and during transition from long-term care into the community (7–9).

Antipsychotic polypharmacy has been associated with a number of deleterious effects. Carnahan and colleagues (10) found an association between antipsychotic polypharmacy and an increase in extra-pyramidal symptoms. In a study by Hashimoto, Uno, Miwa, Kurihara, Tanifuji, and Tensho (11), patients on an antipsychotic polypharmacy regimen reported a higher number of side effects compared to those on monotherapy treatment, including weight gain, dry mouth, and sexual dysfunction. Correll and colleagues (8) found antipsychotic polypharmacy was associated with a significant increase in lipids and a higher prevalence of metabolic syndrome (polypharmacy group: 50.0%; monotherapy group: 34.3%). Some antipsychotic combinations (e.g., ziprasidone and clozapine, sertindole and clozapine) may increase the corrected QT interval (QTc), which increases the risk of ventricular tachycardia and sudden cardiac death (12). The impact of antipsychotic polypharmacy on neurocognitive functioning has been more mixed. Some research has shown a relationship between antipsychotic polypharmacy and decreased neurocognitive performance (13, 14). However, a meta-analysis by Nielsen and colleagues demonstrated no significant difference in cognition between patients on clozapine monotherapy and those prescribed clozapine with a second antipsychotic (15). Lastly, antipsychotic polypharmacy has been associated with an increase in mortality (16).

The adverse sequelae arising from concomitant antipsychotic use have resulted in a bourgeoning field of research investigating correlates of antipsychotic polypharmacy. The literature has identified a number of factors associated with antipsychotic polypharmacy, including younger age, male sex, schizophrenia and psychotic disorders, longer inpatient stay, and greater number of hospital admissions (8, 17). A variety of clinical and medication variables were also found to be related to antipsychotic polypharmacy (e.g., greater illness severity, higher total dose of antipsychotics, treatment with a depot antipsychotic) (8).

Despite the extensive documentation of antipsychotic polypharmacy in numerous clinical scenarios, there has been less research in forensic psychiatric settings. Völlm, Chadwick, Abdelrazek, and Smith examined psychotropic prescribing in a forensic setting and found 62% of patients were prescribed multiple psychotropics (18). Additionally, a small number of studies have investigated the prevalence of antipsychotic polypharmacy in forensic psychiatric settings in Australia and the United Kingdom (prevalence ranges from 11 to 27%) (19–22). To our knowledge, there has not been an investigation into the prevalence of antipsychotic polypharmacy in a forensic psychiatric setting in North America. Additionally, there have been no studies looking at factors related to antipsychotic polypharmacy in forensic patients. This is an important area of investigation, as forensic psychiatric patients are a complex population frequently presenting with long-standing concurrent psychiatric and substance use disorders (23). Complicating the intricacy of their comorbidities, these patients often have limited insight into their illnesses, making them a treatment challenge for mental health clinicians (24). Placing forensic psychiatric patients on an efficacious treatment regimen that improves psychiatric functioning while simultaneously reducing aggression is fundamental to ensure the dual nature of the forensic system is met, in which patient liberties are maximized while maintaining public safety.

The purpose of this study was to gain knowledge of antipsychotic prescribing patterns, determine the prevalence of antipsychotic polypharmacy, and identify factors related to antipsychotic polypharmacy in a forensic psychiatric setting. Forensic psychiatry is a branch of psychiatry that incorporates aspects of both law and psychiatry, operating at the intersection between the mental health and criminal justice systems (25). In Canada, individuals are placed under forensic psychiatric care after a legal finding of not criminally responsible on account of mental disorder (NCRMD) or unfit to stand trial due to a severe mental illness or disability (26). Given the complex concurrent disordered nature of the forensic psychiatric population (including severe mental illness and frequent comorbid substance use and personality disorders) we hypothesize that higher rates of polypharmacy exist in our forensic inpatient sample than many other clinical settings (e.g., community inpatient). In addition to assessing demographic and clinical variables, we opted to include factors relevant to forensic psychiatric care (e.g., index offence, security level, access to the community, and legal status).

## Methods

### Design

The authors employed a cross-sectional design to investigate psychotropic prescribing practices, prevalence of antipsychotic polypharmacy, and factors associated with antipsychotic polypharmacy in a forensic psychiatric hospital. This retrospective chart review received ethics approval from the University of British Columbia and BC Mental Health and Substance Use Services.

### Sample and Setting

The study was conducted at a 190-bed psychiatric hospital (96 high/medium security beds and 94 low security beds) in Western Canada specialized in forensic psychiatric care. Patients were included in the study based on the following criteria: 1) the patients were under a treatment order (i.e., had received a legal designation by the court of NCRMD or unfit to stand trial); 2) had received a prescription or medication review since admission. The above criteria excluded individuals who were at the facility for temporary treatment (e.g., mentally disordered accused awaiting trial or acutely unwell offenders serving a custodial sentence and admitted for psychiatric treatment) or those undergoing forensic assessment for criminal responsibility or fitness to stand trial designations.

### Procedure

Data was collected from pharmacy records and medical charts and entered into an electronic research data capture tool. Pharmacological variables included medication name and class, type of prescription order [i.e., a regular order or *pro re nata* (PRN) order], dose, and route of administration. Demographic (i.e., age, gender, ethnicity, education level), clinical (i.e., primary psychiatric diagnosis, secondary psychiatric diagnoses, length of current hospitalization), and forensic (i.e., most serious index offence, community access, security level, legal status) variables were collected from patient charts.

For the purpose of our study, antipsychotic polypharmacy was defined as two or more concurrent, regular dosage, antipsychotic orders. This definition of antipsychotic polypharmacy is frequently employed in this area of research (18, 27). To determine if a patient was prescribed a high dose of antipsychotics, we converted the prescribed dosage to a percentage of the maximum recommended dose [as identified on the Canadian version of the Prescribing Observatory for Mental Health (POMH) antipsychotic dosage ready reckoner] for each drug and summed the percentages. A patient was considered to be on a high dose of antipsychotic medication if the percentages combined exceeded 100%. This method of calculating high dosages of antipsychotics has been frequently employed in prior research on antipsychotic polypharmacy (28, 29).

### Analysis

Data analysis was conducted using SPSS Version 25.0 for Windows. Descriptive analysis included frequencies, means, standard deviations, and ranges for the reported variables. To test associations between categorical variables, Pearson's chi-squared test or Fisher's exact test (for variables that did not meet assumptions of the chi-squared test) were performed. For continuous variables, the normality of distribution was assessed with the Kolmogorov-Smirnov one-sample test. Both patient age and length of current hospitalization were significantly different from normal distributions. Therefore, Mann-Whitney *U*-tests were used to assess differences between groups for these variables. Statistical significance was set for all analyses at two-tailed *p* < .05.

Demographic, clinical, and forensic variables with a significance of *p* < .05 at the bivariate level were included in a binomial logistic regression model to identify characteristics predictive of exposure to antipsychotic polypharmacy. The dependent variable was presence of antipsychotic polypharmacy. Sample size calculation for the logistic regression was based on guidelines outlined in Peduzzi and colleagues (30), following the formula N=10 k/p. For a model with five predictors, a sample of 91 was required (our sample size of 142 met this requirement).

## Results

### Demographics

Medication and clinical file reviews were conducted for 142 patients who met inclusionary criteria. **Table 1** presents the demographic, clinical, and forensic characteristics of the sample. Overall, the sample was primarily male (94.37%), Caucasian (70.42%), and had a mean age of 40.25 years (*SD* = 13.84). The average length of current hospitalization was 4.82 years (*SD* = 6.34). The majority of patients had a primary diagnosis on the schizophrenia spectrum (83.10%). The most common comorbid psychiatric disorder was a substance use disorder (68.31%). The majority of patients were exempted from criminal responsibility or found unfit to stand trial on a violent index offence against a person, with 30.28% having committed homicide/attempted homicide. At the time of our cross-sectional date, 52.82% of patients resided on low security units and 57.04% had access to the community (which consisted of either staff supervised or unsupervised excursions).

**Table 1:** Demographic, clinical, and forensic characteristics of the sample.

Characteristics	Number (%) *N* = 142
Gender Male Female	134 (94.37) 8 (5.63)
Age (in years) Mean (SD) Range	40.25 (13.84) 19.00–76.00
Ethnicity Caucasian First Nations Asian Other	100 (70.42) 20 (14.08) 12 (8.45) 10 (7.05)
Education level High school not completed High school or more completed	88 (61.97) 54 (38.03)
Length of current hospitalization (in years) Mean (SD) Range	4.82 (6.34) 0.02–36.62
Security level High/medium security Low security	67 (47.18) 75 (52.82)
Access to the community No community access Community access	61 (42.96) 81 (57.04)
Primary psychiatric diagnosis Schizophrenia spectrum/psychotic disorders Mood disorders Neuropsychiatrics disorders Other psychiatric diagnoses	118 (83.10) 4 (2.82) 16 (11.26) 4 (2.82)
Secondary psychiatric diagnoses Psychotic disorders Mood disorders Substance use disorders Neuropsychiatric disorders Personality disorders Other psychiatric diagnoses	14 (9.86) 8 (5.63) 97 (68.31) 40 (28.17) 17 (11.97) 14 (9.86)
Most serious index offence Homicide/attempted homicide/manslaughter Other violent crimes All other crimes	43 (30.28) 75 (52.82) 24 (16.90)
Legal status NCRMD Unfit Involuntary	130 (91.55) 5 (3.52) 7 (4.93)

NCRMD, not criminally responsible on account of mental disorder.

### Psychotropic Prescriptions

Antipsychotics were prescribed to every patient in our sample (see **Table 2**). Regular antipsychotics were prescribed in oral form to 123 patients (86.62%) and in a depot formulation to 69 patients (48.59%). Regular antipsychotics were prescribed in high doses for 57 patients (39.86% of the sample). Of the 57 patients taking high-dose antipsychotics, the majority were on a high-dose regimen as a result of taking two or more antipsychotics concurrently (*N* = 48, 84.21%). For the remaining psychotropic classes, antidepressants were prescribed to 43.66% of patients, followed by mood stabilizers (31.69%), anticholinergics (20.42%), anxiolytics (13.38%), and stimulants (1.41%).

**Table 2:** Frequencies of psychotropics prescribed and associations between types of psychotropics prescribed and exposure to antipsychotic polypharmacy.

Psychotropic	Sample *N* = 142	Monotherapy *N* = 64	Polypharmacy *N* = 78	Chi square (χ2)	P value
Antipsychotics Regular PRN High dose Clozapine Depot	142 (100.00) 119 (83.80) 57 (40.14) 49 (34.51) 69 (48.59)	64 (100.00) 53 (82.81) 9 (14.06) 15 (23.44) 20 (14.08)	78 (100.00) 66 (84.62) 48 (61.54) 34 (43.59) 49 (34.51)	— 0.084 32.977 6.317 14.027	— .772 <.001 .012 <.001
Antidepressants Regular PRN	62 (43.66) 3 (2.11)	23 (35.93) 0 (0.00)	39 (50.00) 3 (3.85)	2.826	.093 .252
Anxiolytics Regular PRN	19 (13.38) 90 (63.38)	5 (7.81) 41 (64.62)	14 (17.95) 49 (62.82)	3.116 0.023	.078 .879
Mood stabilizers Regular PRN	45 (31.69) 0 (0.00)	16 (25.00) 0 (0.00)	29 (37.18) 0 (0.00)	2.409 —	.121 —
Stimulants Regular PRN	2 (1.41) 0 (0.00)	2 (3.13) 0 (0.00)	0 (0.00) 0 (0.00)	—	.201 —
Anticholinergics Regular PRN	29 (20.42) 95 (66.90)	12 (18.75) 34 (53.13)	17 (21.79) 61 (78.21)	0.201 9.986	.654 .002

Antipsychotics were similarly the most commonly prescribed PRN psychotropic (83.80% of the sample). PRN antipsychotics were prescribed in oral form to 83.10% of the sample and in injectable form to 53.52% of the sample. For the remaining PRN psychotropics, anticholinergics were prescribed to 66.90% of patients, followed by benzodiazepines (63.38%) and antidepressants (2.11%). No patients were prescribed a mood stabilizer or stimulant on an “as needed” basis.

#### Frequency of Antipsychotics Prescribed

A total of 167 regular oral antipsychotics were prescribed across the sample. Most oral antipsychotics prescribed were second generation antipsychotics (81.44%). The most common oral antipsychotic was clozapine (28.14%), followed by olanzapine (22.15%) and quetiapine (12.57%). A total of 69 depot antipsychotics were prescribed. First generation antipsychotics made up 50.72% of the depot antipsychotics prescribed. The most frequently prescribed depot antipsychotics were zuclopenthixol decanoate (36.23%), paliperidone palmitate (27.54%), and aripiprazole (11.59%).

A total of 141 oral PRN antipsychotics were prescribed to the sample. The most common oral PRN antipsychotic was loxapine (61.00%), followed by quetiapine (13.48%) and methotrimeprazine (also known as levomepromazine) (12.77%). A total of 83 injectable PRN antipsychotics were prescribed. The most common injectable antipsychotic was loxapine (73.49%), followed by haloperidol (10.84%) and methotrimeprazine (9.64%).

### Antipsychotic Polypharmacy

An antipsychotic polypharmacy regimen was prescribed for 78 patients (54.93%). Of those patients on an antipsychotic polypharmacy regimen, 67 (85.90%) patients were prescribed two concomitant antipsychotics, while 10 (12.82%) patients received three antipsychotics, and only one (1.28%) patient was taking four regular antipsychotics simultaneously. Forty-one different antipsychotic combinations were administered. Eleven of the 41 (26.83%) combinations of antipsychotics involved augmentation of clozapine with another agent. The most common antipsychotic combination was clozapine and risperidone (8.97% of those on an antipsychotic polypharmacy regimen), followed by clozapine and zuclopenthixol, olanzapine and paliperidone, and aripiprazole and clozapine (7.69% for each combination).

Bivariate analysis found that antipsychotic polypharmacy was significantly associated with length of current hospitalization (*U* = 1,949.50, *p* =.025); security level [χ2(1, *N* = 142) = 4.384, *p* =.036]; and having a comorbid personality disorder [χ2(1, *N* = 142) = 5.866, *p* =.015; see **Table 3**]. Other patient characteristics, including age, ethnicity, and primary psychiatric diagnosis were not found to be associated with an antipsychotic polypharmacy regimen. Being placed on an antipsychotic polypharmacy regimen was also associated with a concomitant prescription of PRN anticholinergics [χ2(1, *N* = 142) = 9.986, *p* =.002] and with being on a high dose of antipsychotics [χ2(1, *N* = 142) = 32.977, *p* < .001]. There was also an association between being exposed to an antipsychotic polypharmacy regimen and being prescribed clozapine [χ2(1, *N* = 142) = 6.317, *p* =.012]. There were no associations between an antipsychotic polypharmacy regimen and being prescribed other classes of psychotropic drugs.

**Table 3:** Associations between demographic, clinical, and forensic variables and exposure to antipsychotic polypharmacy.

Characteristics	Monotherapy *N* = 64	Polypharmacy *N* = 78	Test statistic	P value
Gender Male Female	59 (92.19) 5 (7.81)	75 (96.15) 3 (3.85)		.468
Age (in years) Mean (SD) Range	38.80 (13.13) 19.00–69.00	41.44 (14.36) 21.00–76.00	*U* = 2,238.500	.291
Ethnicity Caucasian First Nations Asian Other	42 (65.62) 10 (15.63) 7 (10.94) 5 (7.81)	58 (74.36) 10 (12.82) 5 (6.41) 5 (6.41)	χ2 = 1.528	.676
Education level High school not completed High school or more completed	41 (64.06) 23 (35.94)	47 (60.26) 31 (39.74)	χ2 = 0.216	.642
Length of current hospitalization (in years) Mean (SD) Range	3.57 (4.56) 0.03–23.29	5.85 (7.36) 0.02–36.62	*U* = 1,949.500	.025
Security level High/medium security Low security	24 (37.50) 40 (62.50)	43 (55.13) 35 (44.87)	χ2 = 4.384	.036
Access to the community No community access Community access	30 (46.88) 34 (53.12)	31 (39.74) 47 (60.26)	χ2 = 0.730	.393
Primary psychiatric diagnosis Schizophrenia spectrum/psychotic disorders Mood disorders Neuropsychiatric disorders Other psychiatric diagnosis	53 (82.81) 2 (3.13) 8 (12.50) 1 (1.56)	65 (82.05) 2 (2.56) 8 (11.54) 3 (3.85)		.873
Secondary psychiatric diagnoses Psychotic disorders Mood disorders Substance use disorders Neuropsychiatrics disorder Personality disorders Other psychiatric diagnoses	6 (9.38) 5 (7.81) 44 (68.75) 22 (34.38) 3 (4.69) 5 (7.81)	8 (10.26) 3 (3.85) 53 (67.95) 18 (23.08) 14 (17.95) 9 (11.54)	χ2 = 0.031 χ2 = 0.010 χ2 = 2.218 χ2 = 5.866 χ2 = 0.549	.861 .468 .919 .136 .015 .459
Most serious index offence Homicide/attempted homicide/manslaughter Other violent crimes All other crimes	20 (31.25) 34 (53.13) 10 (15.62)	23 (29.49) 41 (52.56) 14 (17.95)	χ2 = 0.150	.928
Legal status NCRMD Unfit Involuntary	59 (92.19) 2 (3.13) 3 (4.68)	71 (91.03) 3 (3.85) 4 (5.13)	χ2 = 0.071	.965

NCRMD, not criminally responsible on account of mental disorder.

Variables significant at the bivariate level were entered into a logistic regression model to assess their predictiveness of exposure to antipsychotic polypharmacy (see **Table 4**). We opted to not include prescription of PRN anticholinergics or high dose antipsychotic variables into the model as these variables were likely consequences of being on an antipsychotic polypharmacy regimen (e.g., taking an anticholinergic PRN to reduce the side effects of antipsychotics). The model containing five predictors was significant (χ2 = 40.338, *p* < .001). The strongest factor associated with antipsychotic polypharmacy was receiving a depot antipsychotic (OR = 5.71, *p* < .001). This suggests that patients receiving a depot antipsychotic prescription were at least five times more likely to be on an antipsychotic polypharmacy regimen. The use of clozapine (OR = 3.75, *p* =.003) was also associated with treatment with concomitant antipsychotics. Patients who resided on low security units were less likely (OR = 0.36, *p* =.01) than those residing on high/medium security units to be on an antipsychotic polypharmacy regimen. For every year increase in length of hospitalization, there was an increase in odds of being placed on antipsychotic polypharmacy (OR = 1.08, *p* =.03). Having a comorbid diagnosis of a personality disorder failed to reach significance in the model (OR = 3.81, *p* =.10).

**Table 4:** Predictors of exposure to antipsychotic polypharmacy using binomial logistic regression.

Characteristic	B	SE	Wald	df	p	OR	95% CI	
							Lower	Upper
Depot antipsychotic	1.743	.427	16.666	1	<.001	5.712	2.474	13.185
Treatment with clozapine	1.323	.449	8.688	1	.003	3.754	1.558	9.048
Low security	−1.020	.401	6.461	1	.011	0.361	0.164	0.792
Length of hospitalization	0.078	.036	4.580	1	.032	1.081	1.007	1.161
Personality disorder	1.337	.802	2.782	1	.095	3.809	0.791	18.340

## Discussion

This study examined the prevalence and factors associated with antipsychotic polypharmacy in a forensic psychiatric sample. The prevalence of antipsychotic polypharmacy in this sample was higher than the majority of recent documented rates (5, 6). We propose a few hypotheses for the higher rate of antipsychotic polypharmacy compared to the broader literature. First, the high prevalence of antipsychotic polypharmacy may reflect a cohort of patients that have more severe psychopathology and higher clinical needs than patients in other studies (e.g., civil psychiatric inpatients, outpatients, etc.). Forensic patients are considered to be more difficult to treat due to the nature of their often treatment-resistant illnesses and comorbid substance use diagnoses (23, 31). Therefore, there may have been an increased utilization of antipsychotic polypharmacy to manage complex psychiatric symptoms. Second, antipsychotics are often used not only to treat the symptoms of a psychotic illness, but also to reduce aggression (32, 33). This may partly explain why a small subset of patients in our sample who did not have a primary psychotic disorder were also prescribed at least one antipsychotic as part of their treatment regimen. Further, additional antipsychotics may have been prescribed to some patients as part of a risk mitigation strategy. Finally, the legal mandate for forensic institutions to treat and re-integrate patients into the community may have contributed to antipsychotic polypharmacy. There may have been an increased tendency toward multiple antipsychotics in order to manage psychiatric symptoms such that patients will be able to successfully integrate into society. Future research is needed to investigate the clinical justifications for concomitant antipsychotic prescriptions to help uncover the factors that may influence polypharmacy.

The most common antipsychotic combination prescribed was clozapine and risperidone, followed by clozapine and zuclopenthixol, clozapine and aripiprazole, and olanzapine and paliperidone. There is a paucity of evidence available to guide clinicians in choosing antipsychotic combinations that may reasonably be utilized in challenging clinical situations. Some clinical guidelines suggest that, in response to a failed clozapine trial, a second antipsychotic can be added to clozapine (6). However, research investigating the therapeutic benefit of clozapine augmentation is mixed. For example, a randomized controlled trial by Honer and colleagues (34) found no improvement in psychiatric symptoms when clozapine was augmented with risperidone compared to clozapine monotherapy. Additionally, a review by Kroken and Johnsen (35) concluded that there was no concrete evidence supporting the addition of a second antipsychotic agent to clozapine for patients with treatment-resistant schizophrenia. However, a meta-analysis by Taylor, Smith, Gee, and Nielsen (36) found a modest decrease in psychiatric symptom severity with antipsychotic combinations that involved clozapine. In our study, most antipsychotic combinations did not involve the use of clozapine. There is far less evidence available regarding the efficacy of non-clozapine antipsychotic combinations. In fact, the large number of non-clozapine antipsychotic combinations in this study may reflect an underutilization of clozapine in this setting (37). It is possible that public safety considerations at the time of discharge influence a patient's antipsychotic regimen. For example, some hospital patients may be deemed suitable candidates for clozapine treatment prior to discharge, but if insight regarding compliance with daily clozapine dosing and motivation for regular blood work are suboptimal, a prescriber may instead choose to utilize a combination of clozapine and injectable medication or combined injectable antipsychotic regimen. Therefore, diminishing societal risk tolerance for psychiatrically driven violence by forensic mental health service users may be a partial explanation for the underutilization of clozapine, where full compliance with dosing and blood monitoring is essential to mitigate public safety risk. In sum, the evidence for augmenting clozapine with another antipsychotic does not appear to be fully elucidated, and there is a dearth of evidence supporting the use of non-clozapine antipsychotic combinations. There is a strong need for further research investigating the use of varying combinations of antipsychotics.

Given the increasing prevalence of antipsychotic polypharmacy, a number of researchers have investigated methods of reducing this practice. Westaway, Sluggett, Alderman, Procter, and Roughead (38) suggested strategies that educate prescribing clinicians about antipsychotic polypharmacy could be an effective way to reduce polypharmacy. In a cluster randomized controlled trial, Thompson and colleagues (39) provided education workbooks and reminders to clinicians when patients were prescribed multiple antipsychotics. They found a considerable reduction in antipsychotic polypharmacy prescribing in the intervention group compared to the control group. A systematic review by Tani, Uchida, Suzuki, Fuji, and Mimura (40) found interventions to reduce antipsychotic polypharmacy were the most effective when the approach was active (e.g., targeted communication to a physician) compared to passive (e.g., informative lectures about the effects of antipsychotic polypharmacy). The available research suggests that reducing antipsychotic polypharmacy is feasible in a number of clinical circumstances. For example, two studies have found that individuals switched from a polypharmacy to a monotherapy regimen did not demonstrate a significant change in psychiatric symptoms (41, 42). There may be additional benefits derived from reducing antipsychotic polypharmacy. Essock and colleagues (42) found resuming an antipsychotic monotherapy regimen resulted in a decrease in body mass index (BMI). Hori, Yoshimura, Katsuki, Sugita, Atake, and Nakamura (43) investigated changes in cognitive and social functioning of patients switched to an antipsychotic monotherapy regimen and found significant increases in attention and vocational skills compared to a group maintained on a polypharmacy regimen. Overall, the available evidence suggests that with appropriately designed interventions, the majority of patients on an antipsychotic polypharmacy regimen can be safely switched to an antipsychotic monotherapy regimen with no significant deterioration in clinical functioning.

A number of factors were predictive of antipsychotic polypharmacy, including longer duration of hospitalization, residing on a high/medium security unit, receiving depot antipsychotics, and treatment with clozapine. Some of the factors predictive of antipsychotic polypharmacy found in this study are consistent with the broader literature. For example, duration of hospitalization has been a well-documented predictor of antipsychotic polypharmacy in many clinical settings (27). Residing on a high/medium security unit may be predictive of antipsychotic polypharmacy due to the treatment complexities of these patients, including aggressive behaviors and severe psychopathology (e.g., treatment-resistant psychosis). There may also be greater comfort among mental health clinicians to trial antipsychotic polypharmacy on a secure unit where patients are closely monitored. Some factors that are frequently related to antipsychotic polypharmacy in the literature (e.g., age, gender, ethnicity, primary psychiatric diagnosis) were not found to be associated with antipsychotic polypharmacy in our sample. For example, age may not be related to antipsychotic polypharmacy as medication regimens in forensic settings need to be appropriate and responsive to both symptom severity and level of violence risk, neither of which may be correlated with age. In some cases (e.g., gender, primary psychiatric diagnosis) there was likely a lack of power to observe any differences. Interestingly, many forensic factors (e.g., access to the community, most serious index offence, and legal status) were not related to antipsychotic polypharmacy. There are only a few studies that have assessed the relationship between forensic factors and antipsychotic polypharmacy. In one study, Yip and colleagues (44) found a history of violence was significantly associated with antipsychotic polypharmacy. In another study, Connolly and Taylor (45) identified an association between having a forensic history and antipsychotic polypharmacy. Additional research with forensic samples is needed to further clarify the relationship between these variables and antipsychotic polypharmacy. Overall, the factors predictive of antipsychotic polypharmacy (e.g., clozapine use, depot antipsychotics) suggest these individuals are long stay patients who may present a greater treatment difficulty.

It is important to frame our results in the context of our limitations. First, due to the cross-sectional nature of the study, we were unable to determine if some instances of antipsychotic polypharmacy were due to cross-tapering of antipsychotics or if they were being used temporarily for another clinical indication. Second, some factors including duration of psychiatric illness and amount of psychiatric hospitalizations were not included in our study and would be important variables to consider in further research. Third, our study had a small percentage of females in this study. This limitation is in part due to the small number of female forensic hospital patients. Future research should strive to include a larger number of female patients to understand if there is a relationship between gender and antipsychotic polypharmacy in forensic settings. Additionally, our study sample did not include other categories of patients receiving forensic psychiatric care (e.g., forensic psychiatric outpatients, patients under court ordered assessment, individuals receiving temporary treatment from jail). Further research is needed to understand the prevalence and factors associated with antipsychotic polypharmacy in these distinct groups.

## Conclusion

The current study provided new information regarding the demographic, clinical, and forensic factors associated with antipsychotic polypharmacy treatment in a forensic psychiatric sample. The results suggested that the patients most likely to receive antipsychotic polypharmacy were long stay patients that presented with greater treatment complexities. The knowledge gained from this study can be used to inform healthcare leadership of current treatment strategies and guide targeted interventions to identify antipsychotic polypharmacy and encourage clinicians to evaluate instances of concomitant antipsychotic use in clinical practice. Future research should strive to further our understanding regarding clinical justifications for prescribing multiple concomitant antipsychotics. Additionally, there are still areas within forensic psychiatry that need further investigation, including forensic psychiatric outpatients and those receiving court ordered assessments. The contribution of patient insight, public risk tolerance levels, and treatment provider attitudes in medication decision making processes are important, yet unexplored variables in forensic psychiatric practice. The impact of these variables as possible drivers for polypharmacy practices in forensic psychiatric hospital and community settings constitute a possible further extension of the present study.

## Data Availability Statement

The raw data supporting the conclusions of this article will be made available by the authors, without undue reservation, to any qualified researcher.

## Ethics Statement

The studies involving human participants were reviewed and approved by the Clinical Research Ethics Board, University of British Columbia. Written informed consent for participation was not required for this study in accordance with the national legislation and the institutional requirements.

## Author Contributions

CF and JB designed the research protocol. CF collected the data and undertook data analysis. CF wrote the initial draft of the paper. JB provided edits to the paper. Both CF and JB reviewed the final draft of the paper and have approved this manuscript.

## Conflict of Interest

The authors declare that the research was conducted in the absence of any commercial or financial relationships that could be construed as a potential conflict of interest.
